# A Peroxidase-linked Spectrophotometric Assay for the Detection of Monoamine Oxidase Inhibitors

**Published:** 2016

**Authors:** Kangkang Zhi, Zhongduo Yang, Jie Sheng, Zongmei Shu, Yin Shi

**Affiliations:** a *School of Life Science and Engineering, Lanzhou University of Technology, Lanzhou 730050, PR China, *; b *Key Lab of New Animal Drug Project, Gansu Province; Key Lab of Veterinary Pharmaceutical Development, Ministry of Agriculture; Lanzhou Institute of Husbandry and Pharmaceutical Sciences of CAAS, Lanzhou 730050, PR China.*

**Keywords:** Monoamine oxidase inhibitors, Amine substrate, Spectrophotometric Assay, HPLC

## Abstract

To develop a new more accurate spectrophotometric method for detecting monoamine oxidase inhibitors from plant extracts, a series of amine substrates were selected and their ability to be oxidized by monoamine oxidase was evaluated by the HPLC method and a new substrate was used to develop a peroxidase-linked spectrophotometric assay. 4-(Trifluoromethyl) benzylamine (11) was proved to be an excellent substrate for peroxidase-linked spectrophotometric assay. Therefore, a new peroxidase-linked spectrophotometric assay was set up. The principle of the method is that the MAO converts 11 into aldehyde, ammonia and hydrogen peroxide. In the presence of peroxidase, the hydrogen peroxide will oxidize 4-aminoantipyrine into oxidised 4-aminoantipyrine which can condense with vanillic acid to give a red quinoneimine dye. The production of the quinoneimine dye was detected at 490 nm by a microplate reader. The ⊿_OD_ value between the blank group and blank negative control group in this new method is twice as much as that in Holt’s method, which enables the procedure to be more accurate and avoids the produce of false positive results. The new method will be helpful for researchers to screening monoamine oxidase inhibitors from deep-color plant extracts.

## Introduction

Monoamine oxidase (MAO) is a mitochondrial enzyme that catalyzes the oxidative deamination of biogenic amines and neurotransmitters (1). It exists as two isozymes, MAO-A and MAO-B, differing in tissue localization, substrate preference, and inhibitor selectivity. MAO plays an important role in the central nervous system and peripheral organs (2, 3). MAO-A inhibitors are used clinically as antidepressants and anxiolytics, while inhibition of MAO-B are used for reduction of the progression of Parkinson’s disease and symptoms associated with Alzheimer’s disease (4). 

**Scheme 1:**
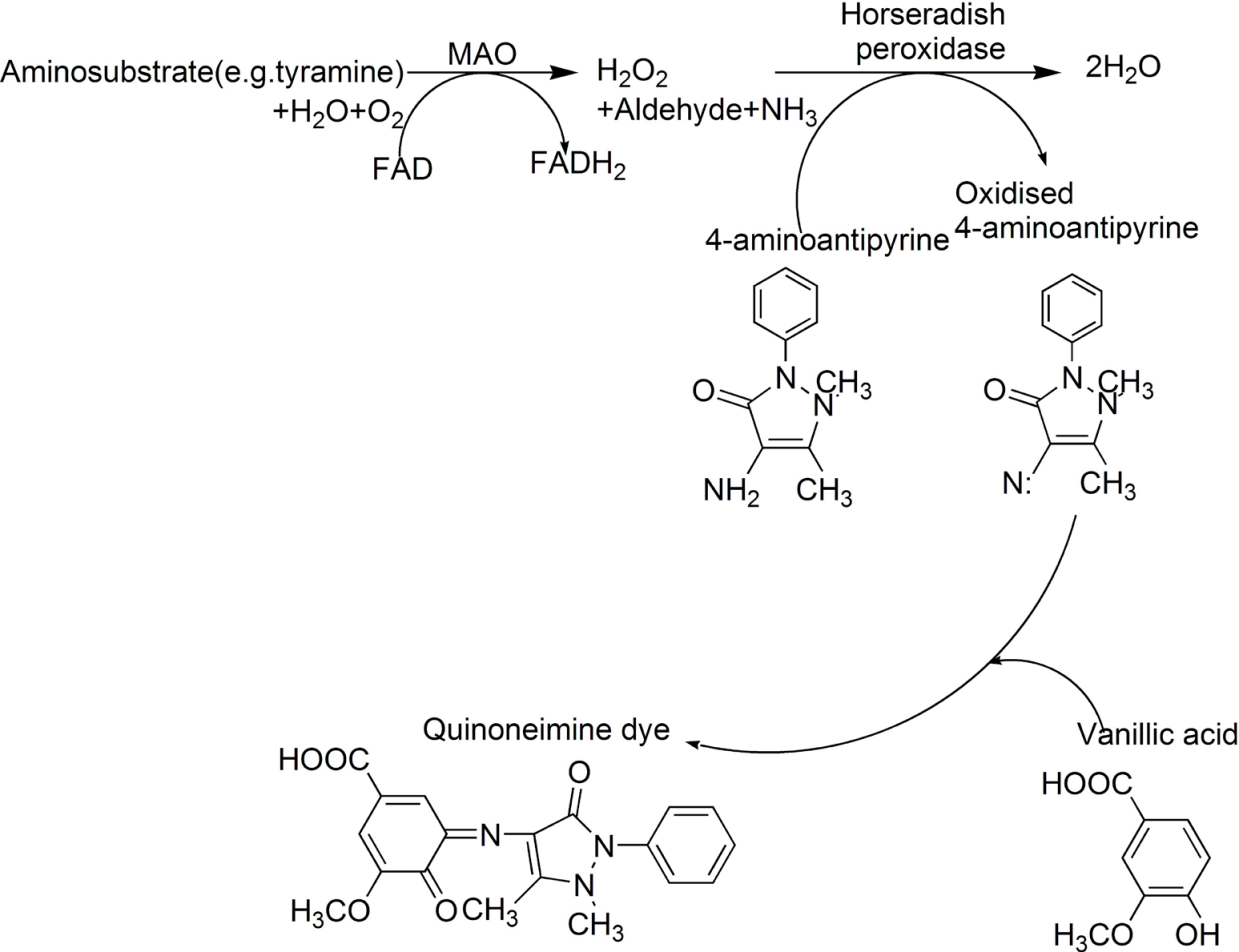
Scheme for peroxidase-linked spectrophotometric monoamine oxidase (MAO) inhibitor assay modified from Holt *et al*

The pharmacological and therapeutic importance of MAO inhibitors（MAOIs） necessitates the development of methods to identify natural MAOIs effectively and accurately in biotic resources. Holt's method is a classical spectrophotometric method in detecting MAOIs (5, 6). The basic principle of this method is that the MAO converts aminosubstrate (RCH_2_NH_2_, often tyramine as substrate) into aldehydes (RCHO), amine (NH_3_) and hydrogen peroxide. The following hydrogen peroxide that produced by the rate determining step oxidizes 4-aminoantipyrine in the presence of peroxidase. The oxidized 4-aminoantipyrine condenses with vanillic acid to give a red quinoneimine dye (Scheme 1). The optical density (OD) values of the quinoneimine dye are detected at 490 nm by a microplate reader. However, for this method, when using rat liver homogenate as enzymic source (often be adopted in many literatures) and tyramine as substrate, the difference value of optical density (⊿_OD_) between the blank group (substrates are present and no inhibitors exist) and blank negative control group (substrates are absent and no inhibitors exist) is small (about 0.2) which makes the procedure be inaccurate and produces many false positive results. Especially, when using this method to detect MAOIs in crude plant extracts, due to the color of extracts, the OD values of experiment groups are very closed to those of corresponding experiment controls (substrates are absent, this groups are set up to deduct sample’s background) which make the ⊿OD values be very small (usually, about 0.05-0.1). So, in sometime, if calculating inhibitory rate of test samples with various concentrations, experimenters will obtain the confusing results. In other words, samples with low concentration show high inhibitory rate while samples with high concentration have low inhibitory rate. Hence, the accuracy and repeatability of this method are not very satisfactory. On the other hand, some substrates, such as tyramine also can condense with the oxidized form of 4-aminoantipyrine to give a colored compound which will deplete a part of aminosubstrate (7). Therefore, new substrates for the enzyme reaction were focused on this study in order to develop a new spectrophotometric method to replace the current methods. 

In this paper, a series of aminosubstrates (1–26) (Table 1) were selected and their ability to be oxidized by MAO were evaluated. Compound 11 was found to be a new candidate substrate for monoamine oxidase, which could be oxidized easily by monoamine oxidase. Finally, using 11 as a new substrate, with a mixture solution of 4-aminoantipyrine, peroxidase and vanillic acid as a chromogenic agent, and iproniazid (a non-selective inhibitor of MAO), clorgyline (a selective inhibitor of MAO-A) and pargyline (a selective inhibitor of MAO-B) as a standard solution for the measurement of IC_50 _(8-10), a new peroxidase-linked spectrophotometric method was established.

**Table 1:** Information of the candidate substrates and their oxidation yield by MAO

**No.**	**Name** (**purity)**	**CAS register number**		**Maximum (nm)**	**Oxidation yield (%)**
1	Tryptamine hydrochloride (98%)	343-94-2		280 nm	24.93%
2	Tyramine (98%)	51-67-2		274 nm	39.43%
3	2-Thiopheneethylamine (98%)	30433-91-1		233 nm	-
4	4-Hydroxy-3-methoxybenzylamine hydrochloride (99%)	7149-10-2		279 nm	45.91%
5	3, 4-Dimethoxyphenethylamine (%)	120-20-7		278 nm	14.03%
6	4-Methoxyphenethylamine (97%)	55-81-2		275 nm	0.00%
7	1-Naphthylmethylamine (98%)	118-31-0		280 nm	49.50%
8	3-Phenyl-1-propylamine (98%)	2038-57-5		257 nm	30.89%
9	4-Fluorobenzylamine (99%)	140-75-0		262 nm	19.92%
10	2-(p-Tolyl) ethylamine (97%)	3261-62-9		263 nm	11.01%
11	4-(Trifluoromethyl) benzylamine (98%)	3300-51-4		262 nm	98.80%
12	Furfurylamine (99%)	617-89-0		276 nm	-
13	2-Phenethylamine (98%)	64-04-0		257 nm	24.77%
14	3-Methoxybenzylamine (98%)	5071-96-5		273 nm	74.27%
15	4-Nitroaniline (99%)	100-01-6		383 nm	0.00%
16	2-Chlorobenzylamine (98%)	89-97-4		266 nm	13.74%
17	3-Hydroxy-4-methoxyphenethylamine hydrochloride (99%)	645-33-0		279 nm	55.65%
18	3-(Trifluoromethyl) benzylamine (98%)	2740-83-2		262 nm	87.88%
19	2-(Trifluoromethyl) benzylamine (98%)	3048-01-9		262 nm	14.94%
20	2-Methoxybenzylamine (97%)	6850-57-3		273 nm	10.97%
21	4-Methoxybenzylamine (98%)	2393-23-9		272 nm	86.30%
22	Benzylamine (99%)	100-46-9		256 nm	43.66%
23	N-Benzylmethylamine (97%)	103-67-3		256 nm	28.70%
24	N, N-Dimethylbenzylamine (99%)	103-83-3		261 nm	0.00%
25	2-Fluorobenzylamine (98%)	89-99-6		262 nm	32.09%
26	2-Bromobenzylamine (98%)	3959-05-5		267 nm	0.00%

## Experimental

*Chemicals*

Clorgyline was purchased from Sigma (St. Louis, MO, USA). Pargyline·HCl, iproniazid, vanillic acid, 4-aminoantipyrine, peroxidase (typeⅡ, from horseradish), and aminosubstrates (1-26) were purchased from Aladdin-Reagent Co. Ltd (Shanghai, China) and J&K Scientific Ltd (Beijing, China). All solvents used were of analytical grade. 

*Equipments*

The chromatographic system consisted of a Waters 1525 Binary Pump (Waters, USA), a Waters 2996 Photodiode Array Detector (Waters, USA). The photodiode array detector (PAD) was set at 200–400 nm. A column of Polaris C18 (250 mm × 4.6 mm i.d., 5 μm) (Metachem, Ventura, CA) was used and the column temperature was set at 30 ℃. The injection volume was 10 μL for each. The HPLC Breeze Software (Waters Corp., Milford, USA) was employed for data acquisition and processing, run under Windows XP (Microsoft, Redmond, USA)

*Preparation of rat liver homogenate*

The male wistar rats (250 ± 20 g) were purchased from Lanzhou University (China) and maintained in accordance with the Guidelines for Animal Care and Use of Laboratory Animals, Lanzhou University, China. Wistar rats were euthanized by cervical dislocation, and livers were quickly removed to wash in ice-cold potassium phosphate buffer (0.2 M, pH 7.6), and stored at -80 °C. The liver tissue (5 g) was homogenized (1:20, w/v) in 0.3 M sucrose at 4 °C. After centrifuging homogenate at 1000 × g for 10 min, the supernatant was further centrifuged at 1200 × g for 15 min. Finally, the supernatant was centrifuged once more at 10000 × g (30 min) to obtain a crude mitochondrial pellet. The pellet was resuspended in 4 mL 0.2 M phosphate buffer (pH 7.6) which was used as enzyme source. Total protein concentration was measured by the method of Bradford (11) and adjusted with buffer (0.2 M; pH 7.6) to 0.5 mg protein per mL (stock solution), which aliquots of 1 mL were stored at −80 °C until required. 

*Preparation of working solution*

Aminosubstrates (1-26) working solution were respectively prepared in potassium phosphate buffer (0.2 M, pH 7.6) at a concentration of 2.5 mM. The chromogenic solution was prepared by mixture of 1 mM vanillic acid (Sigma), 0.5 mM 4-aminoantipyrine, and 4 U/mL peroxidase in potassium phosphate buffer (0.2 M, pH 7.6). The iproniazid standard solution, clorgyline standard solution and pargyline standard solution were respectively serially diluted with potassium phosphate buffer (0.2 M, pH 7.6) to give final concentrations from 25 to 5 μg/mL (six dilutions 90, 72, 54, 36, 27, 18 μM, for iproniazid), 30 to 1 nM (six dilutions 30, 20, 10, 5, 2.5, 1 nM , for clorgyline) and 500 to 25 nM (six dilutions 500, 400, 200, 100, 50, 25 nM, for pargyline). Above solutions were freshly prepared and stored for periods of up to one week at the temperature of 4 °C. Five enzyme working solutions, at the concentrations of 0.17, 0.20, 0.25, 0.33, 0.5 mg protein/mL, respectively, were obtained from the stock solution by an appropriate dilution with potassium phosphate buffer (0.2 M, pH 7.6).

*Evaluation of the ability of substrates to be oxidated by MAO*

The maximum absorption wavelength of substrates (1-26) was determined by a Cary 50 UV–VIS spectrophotometer. Diffuse light transmittance measurements were performed in the 200-800 nm wavelength range. Graphs were recorded with a computer connected to a spectrophotometer, and a data of light transmittance percentage per nanometer was obtained (Table 1).

Oxidation of substrates was analyzed with HPLC. A 100 μL aliquot of a solution of enzyme (final concentration 0.5 mg protein/mL) was placed in a 1.5 mL centrifuge tube. The reaction was initiated by the addition of 400 μL of 2.5 mM tested substrate solution. After incubating at 37 °C for 90 min in a homothermal incubator, the reaction was terminated by the addition of 20 μL of 10 mM iproniazid. This reaction mixture was used in HPLC quantitative analysis. A blank which contained the corresponding volume of buffer in place of enzyme solutions was used to calculate aminosubstrates turnover.

The unreacted substrate was analyzed by high-performance liquid chromatography (HPLC). The solution of substrate oxidized by MAO was filtered through a cellulose acetate membrane filter (0.45 μm, Anpu Co, Shanghai, China) prior to HPLC analysis. An aliquot of the filtrate (10 μL) was injected into a Polaris C18 column and eluted with a mobile phase containing methanol-water-triethylamine-HCl (15-50: 85-50: 0.5: 0.36, v/v/v/v). The operation time was programmed from 10 to 30 min with a flow-rate of 1.0 mL/min. The analysis was carried out at 30 °C and monitored from 200 to 400 nm (shown in Table 1). The quantitative analysis of oxidation yield was achieved by peak area normalization measurements. The oxidation yield of each compound was contrasted against tyramine (2). 

*Screening new substrates for monoamine oxidase peroxidase linked assay*

Compounds 4, 7, 11, 14, 17, 18, 21 and 22, as candidate substrates, were evaluated to establish new monoamine oxidase peroxidase linked assay. The determination was made in 96-well microplate (Corning Costar Corp., Cambridge, MA). Each test well contained 120 μL candidate substrate (compounds 2, 4, 7, 11, 14, 17, 18, 21 or 22, respectively), 40 μL chromogenic solution, 40 μL enzyme solution (0.5 mg protein/mL) and 40 μL buffer. The control well was set up by adding 120 μL buffer instead of 120 μL candidate substrate. After incubating at 37 °C for 90 min, the OD values were read at 490 nm immediately. The difference values of optical density (⊿_OD_) between the tested substrates group and corresponding control groups were calculated. Each ⊿_OD_ of candidate substrates was compared with those of 2 (a substrate used in Holt’s method (5))

*Monoamine oxidase peroxidase linked assay*

The assay was carried out in the 96-well microplates according to the process modified by Holt’s *et al*. (1997). Briefly, 40 μL enzyme solution (0.5 mg protein/mL), 40 μL of sample solution (preincubation 37 °C; 20 min) and 40 μL chromogenic solution were mixed. Reaction was initiated by the addition of 120 μL of 5 mM 11 and then was incubated at 37 °C for 90 min. After that, OD values were measured in a 96-well plate reader at 490 nm immediately. A blank negative control was set up by adding 120 μL buffer instead of 120 μL of 5 mM 11. Blanks were set up by adding 40 μL buffer solutions instead of 40 μL sample solution. The Sample control was set up by adding 120 μL buffer solutions instead of 120 μL substrate solution in order to deduct sample background. All reactions were carried out quintic. Inhibition rate (%) was calculated by the following equation:

$\mathrm{Inhibition rate\%}=\frac{\left(\mathrm{Blank}-\mathrm{Blank negative control}\right)-(\mathrm{Sample}-\mathrm{Sample control})}{\left(\mathrm{Blank}-\mathrm{Bkank negative control}\right)}\times 100$

equation 1

Six standard sample solutions (iproniazid, a non-selective inhibitor of MAO) at concentration of 90, 72, 54, 36, 27, 18 μM were tested respectively to calculate the IC_50_ values. The IC_50_ values were calculated using Grafit 5 (© Erithacus Software Limited). 

The selectivity of known inhibitors against MAO-A and MAO-B were also evaluated in this new assay. In order to measure the selectivity of known inhibitors against MAO-A, an enzyme solution (0.5 mg protein/mL) was pre-incubated (37 °C; 30 min) with a same volume pargyline (500 nM, a selective inhibitor of MAO-B) to entirely inhibit MAO-B, and for the selectivity of MAO-B, vice versa. After the enzyme was pre-incubated, the IC_50_ values of clorgyline against MAO-A and pargyline against MAO-B were determined respectively according above method.

## Results and Discussion

*Evaluation of the ability of substrates to be oxidated by MAO*

A total of 26 compounds (Table 1) were purchased as the substrates for MAO assay. The maximum absorption wavelength (λ_max_) of substrates (1-26), which would be used in HPLC analysis, was determined by a UV-VIS scan. With the λ_max_ data in hand, the ability of substrates (1-26) to be oxidized by MAO was evaluated by the HPLC method and the yield of oxidation of each substrate was calculated (Table 1). In this experiment, the enzyme solution concentration, the substrate concentration and the reaction time were kept the same for all substrates. This made the results easily comparable. The results showed that 4, 7, 11, 14, 17, 18, 21 or 22 could be oxidized more easily than tyramine (2, used in Holt’s method (1997)) and 1, 5, 8, 9, 10, 13, 16, 19, 20, 23 and 25 could only be oxidized slightly, while 6, 15, 24 and 26 could not be oxidized at all by MAO. In addition, compounds 3 and 12 were not evaluated due to being unstable and self-oxidized.

**Figure 1:**
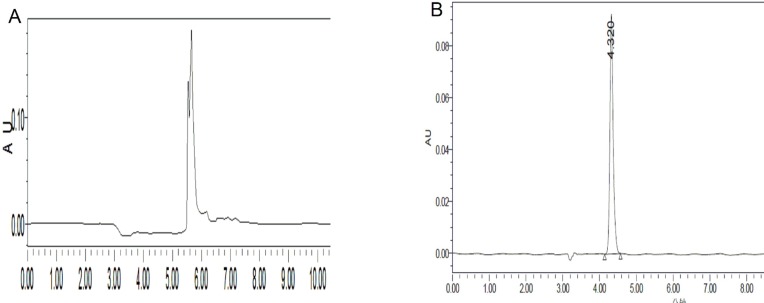
Adding TEA to the mobile phase improves peak shape. A: no TEA B: TEA was adding to mobile phase

In HPLC analysis, there were two aspects needed to be concerned: (1) The maximum absorption wavelength of substrates must be measured at appropriate concentration, because the Beer–Lambert law tends to break down at very high concentrations. The main reason, however, is as following: At high concentrations, the molecules are close to each other and tend to interact with each other. This interaction will probably change several properties of the molecule, such as intermolecular forces which will change the molar absorbtivity (12). Based on our experimental, the appropriate concentration of substrates should be at 0.01 M or less. (2) Acidic silanols on the surface of silica gel stationary phase can form ion-exchange sites that maybe interact with aminosubstrates (basic compounds) which will cause peak tailing when aminosubstrates are analyzed in HPLC apparatus eluting with methanol-water. The solution to peak tailing is to add a competing amine into the mobile phase. In our experiment, triethylamine (TEA) was chosen for this purpose, because it can easily interact with silanols which will repel the reaction of candidate aminosubstrates with stationary phase (13-15) Figure 1 is an example of how TEA improving peak shape. About 0.5% TEA is sufficient for most applications. Because stationary phase is not tolerant for much alkali, 0.36% of HCl was selected to add to mobile phase.

*Screening new substrates for monoamine oxidase peroxidase linked assay*

Compounds 2, 4, 7, 11, 14, 17, 18, 21 or 22, because of their excellent oxidation yield by MAO, were further studied. The ⊿_OD_ values between blank group and blank negative control group of each substrates were listed in Table 2. The results showed that compound 11 have twice higher ⊿_OD_ values than 2 which is a substrate currently widely used (5, 16, 17). Compounds 7, 14, 18, 21 and 22 have approximately same ⊿_OD_ values with 2, while the ⊿_OD_ values of compounds 4, 17 are very small. Based on this result, a new monoamine oxidase peroxidase linked assay was developed using 4-(Trifluoromethyl) benzylamine (11) as the substrate.

**Table 2:** ⊿_OD_ values of substrates 2, 4, 7, 11, 14, 17, 18, 21 and 22.

**Substrates**	**⊿**_OD_ ** values**
2 (used in Holt’s method)	0.198±0.004
4	0.052±0.002
7	0.216±0.003
11	0.425±0.003
14	0.273±0.004
17	0.033±0.003
18	0.284±0.002
21	0.278±0.005
22	0.306±0.003

*Monoamine oxidase peroxidase linked assay*

**Figure 2:**
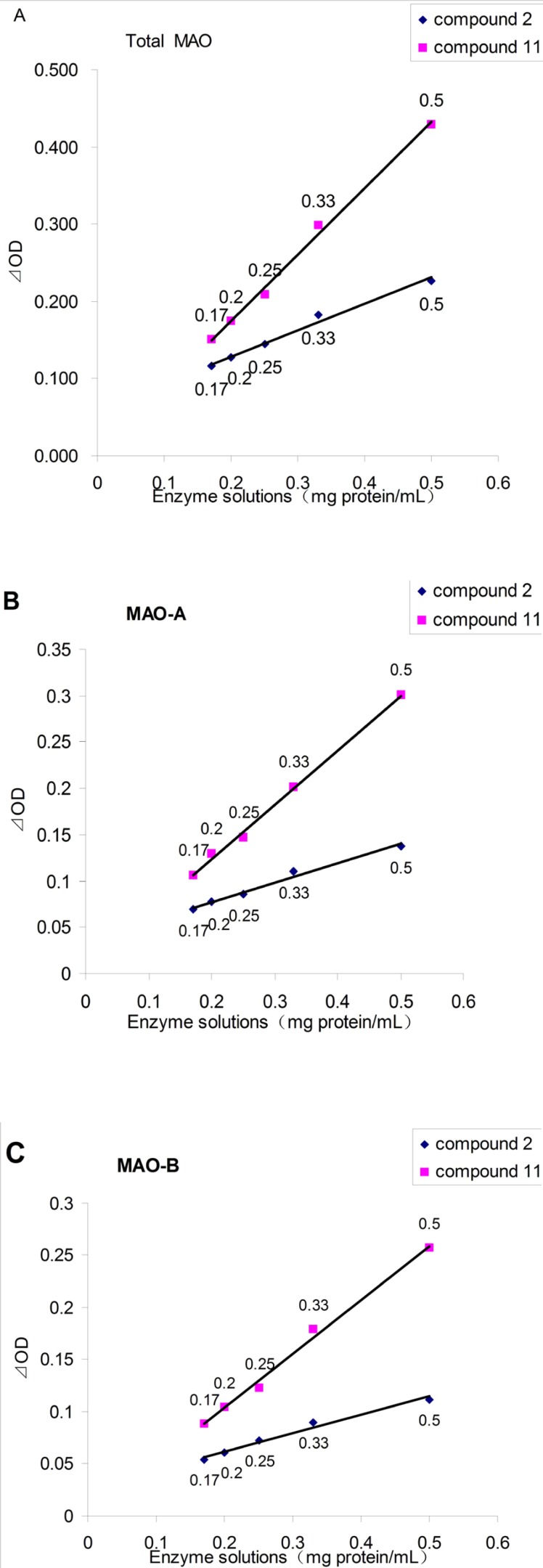
The effect of enzyme solutions concentration on the ⊿_OD_, as assayed by the spectrophotometric assay. A: for Total MAO; B: for MAO-A (add pargyline to inhibit MAO-B); C: for MAO-B (add clorgyline to inhibit MAO-A).

Figure 2 showed the application of the monoamine oxidase peroxidase linked assay of the new substrate (4-(Trifluoromethyl) benzylamine, 11) and a existing substrate (tyramine, 2). In all cases, the ⊿_OD_ values between the blank group and blank negative control group were proportional to the enzyme concentration. It also showed that the ⊿_OD_ values of using 11 as substrate were always larger than those of 2. When the concentration of enzyme solution was 0.5 mg/mL, the ⊿_OD_ values of using 11 as substrate were twice as much as that of compound 2, which would dramatically enhance the accuracy and repeatability, and also reduce false positive results.

**Figure 3:**
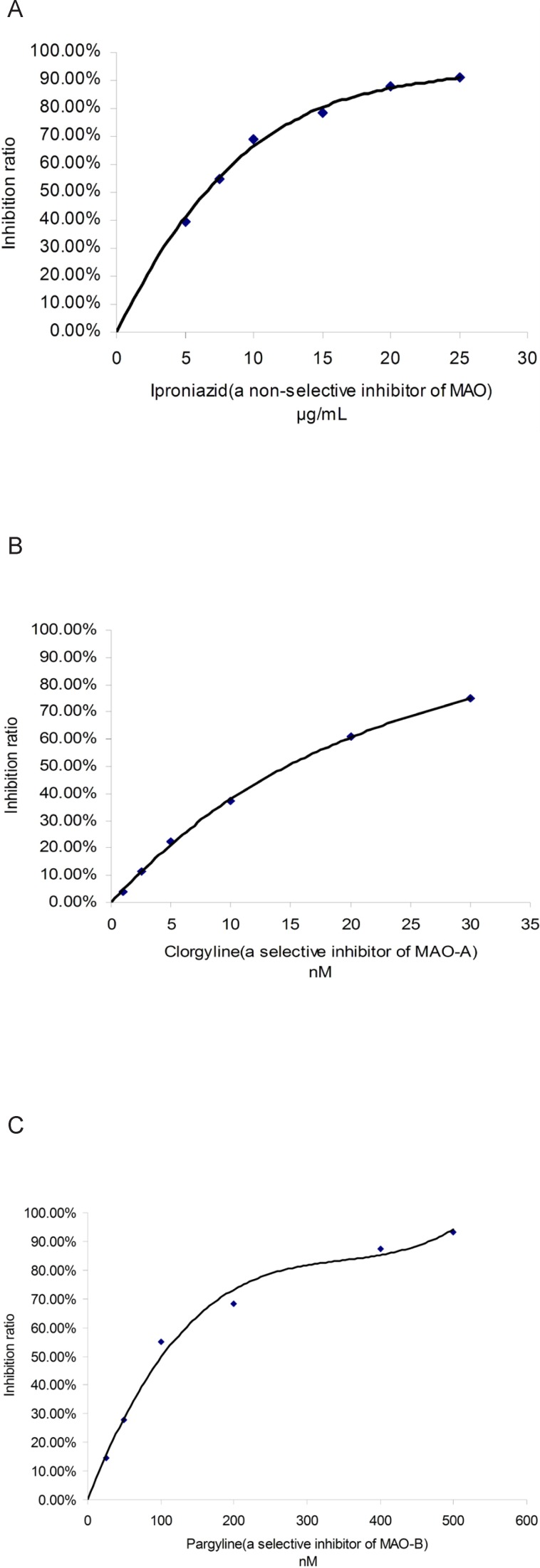
Inhibitory curve of three known inhibitors against MAO. A: iproniazid against total MAO; B: clorgyline against MAO-A; C: pargyline against MAO-B

Three known MAO inhibitors, iproniazid (a non-selective inhibitor of MAO), clorgyline (a selective inhibitor of MAO-A) and pargyline (a selective inhibitor of MAO-B), were selected to confirm the effectiveness of this spectrophotometric method. This three compounds showed MAO, MAO-A and MAO-B inhibitory activity with a dose-dependent behavior, respectively (shown in Figure 3). The IC_50_ values of iproniazid against total MAO, clorgyline against MAO-A and pargyline against MAO-B were 4.02 ± 0.06 μM, 1.96 ± 0.09 nM and 13.40 ± 0.16 nM, respectively which were consistent with those reported in literature (18-20).

Total alkaloid extract of *Coptis chinensis* was also analyzed by this new assay since the plant was reported to contain berberine, jatrorrhizine and palmatime chorides, three potent MAO inhibitors (21). The IC_50_ value (7 μg/mL) of this extract was easily determined by this new assay. But when using Holt’s method (tyramine as subtrate), we found it is difficult, due to poor dose-dependent relationship of concentration and inhibition rate observed. In Holt’s method, the deep color of extract, together with small ⊿_OD _values between blank group and blank negative group, caused the OD value of the sample group was very closed to that of sample control group (sample background). For this, the ⊿_OD_ between sample group and sample control group was very small (just about 0.05-010). Such tiny ⊿_OD_ would make dose-dependent relationship not easily be observed. Of course, if experimenters do it carefully and do many repeat, the IC_50_ of this deep color extract will be also available. But, it will waste a lot of time. In our previous paper (22), we had tested the MAO-inhibition activity of this extract by Holt’s method and the result was consistent with current result.

Up to now, there are several assays that have been used to detect the MAO activity, including Fluorescence assay (23), HPLC assay (24, 25), Proteoliposome Capillary Electrophoresis assay (26), Radiochemical assay (27) and Peroxidase-linked Spectrophotometric Assay (5, 17), etc. The former four methods are either cockamamie for operating, or harmful for bodies, or relying costly apparatus which are not very suitable to widely screen inhibitors from biotic resources. In virtue of easy operability, timesaver and high-throughput, the Peroxidase-linked Spectrophotometric Assay is a favorite method for researchers of natural medicine. However, the current assay has a great disadvantage. That is the ⊿_OD_ value between the blank group and blank negative control group is very small which makes the procedure be inaccurate and causes many false positive results or omits some valuable bio-active extracts. Especially, when using current assay to screening MAO inhibitors from plant extracts with deep-color, researchers usually can not observe the dose-dependent relationship. In our developed assay, with 4-(Trifluoromethyl) benzylamine as substrate, the above-mentioned defect can be improved and the ⊿_OD_ values are satisfactory (about twice as much as Holt’s method).

However, there was a weak point when vanillic acid and 4-aminoantipyrine were employed as reagents. A false-positive result might appear due to some compounds reaction with the oxidized form of 4-aminoantipyrine to give a colored compound, which could be tested by setting up a control experiment with absence of vanillic acid.
